# Exploring plant volatile-mediated interactions between native and introduced plants and insects

**DOI:** 10.1038/s41598-022-18479-z

**Published:** 2022-09-14

**Authors:** Evans Effah, Logan Svendsen, D. Paul Barrett, Andrea Clavijo McCormick

**Affiliations:** College of Sciences, Massey University, Tennent Drive, Palmerston North, 4474 New Zealand

**Keywords:** Ecology, Behavioural ecology, Biodiversity, Community ecology, Conservation biology

## Abstract

In invasion scenarios, native and introduced species co-occur creating new interactions and modifying existing ones. Many plant–plant and plant–insect interactions are mediated by volatile organic compounds (VOCs), however, these have seldom been studied in an invasion context. To fill this knowledge gap, we explored some interactions mediated by VOCs between native and introduced plants and insects in a New Zealand system. We investigated whether a native plant, *Leptospermum scoparium* (mānuka), changes its volatile profile when grown adjacent to two European introduced plants, *Calluna vulgaris* (heather) and *Cytisus scoparius* (Scotch broom), in a semi-field trial using potted plants without above- or below-ground physical contact. We also investigated the influence of plant cues on the host-searching behaviour of two beetles, the native *Pyronota festiva* (mānuka beetle), and the introduced biocontrol agent *Lochmaea suturalis* (heather beetle), by offering them their host-plant and non-host volatiles versus clean air, and their combination in a Y-tube olfactometer. As a follow-up, we performed preference/feeding tests in Petri dishes with fresh plant material. Results of the semi-field experiment show a significant reduction in green leaf volatiles, sesquiterpenes and total volatile emissions by mānuka plants neighbouring heather. In the Y-tube assays, the native beetle *P. festiva* performed poorly in discriminating between host and non-host plants based on plant volatile cues only. However, it performed relatively well in the Petri dish tests, where other cues (i.e., visual, gustatory or tactile) were present. In contrast, the introduced beetle *L. suturalis* showed high host-specificity in both Y-tube and Petri dish assays. This study illustrates the importance of VOCs in mediating interactions between introduced and native species, suggesting that invasive plants can disrupt native plants’ communication and affect the host-searching behaviour of native insects. It also reinforces the relevance of regular host testing on introduced weed biocontrol agents to avoid unwanted host shifts or host-range expansion.

## Introduction

The introduction and establishment of organisms into new habitats is increasing globally with devastating consequences for native biodiversity and ecosystems^1,2^. Invasive species co-exist with one another and with natives creating new interactions and modifying existing ones^3–5^, with positive, negative or neutral consequences. Invasive plants modify the environments they colonise primarily by altering soil properties, microclimate, out-competing native counterparts, and displacing animals (mostly insects) that rely on them^6–9^. Chemically, invasive plants can alter the complexity of invaded habitats by releasing secondary metabolites mainly through root exudates and airborne emissions. A recent publication exploring the allelopathic potential (i.e., the production of chemicals by a plant species that can affect the growth, survival, development or reproduction of neighbouring organisms) of 524 invasive plants shows the majority of these plants produce allelochemicals with the potential to affect native species^10,11^. However, the databases used for this study mainly focus on allelopathic impacts caused through root exudates^12,13^, whereas the role of airborne emissions (volatile organic compounds) in invasion scenarios remains largely unexplored.

Volatile organic compounds (VOCs) are plant natural products with low molecular weight and high vapour pressure at room temperatures. Based on their biosynthetic origin they can be assigned to different classes including terpenoids, phenylpropanoids/benzenoids, fatty acid derivatives (green leaf volatiles) and amino acid derivatives, as well as others not represented in these major classes^14^. Volatile organic compounds are species-specific but also responsive to biotic (e.g., herbivore and pathogen attack) and abiotic factors (e.g., temperature, UV radiation and drought), making them valuable cues about a plant’s identity and state for surrounding organisms^15^. Plant VOCs play an important role in plant–insect interactions by mediating host location and acceptance by pollinators, herbivores and their natural enemies^16,17^. Volatile organic compounds also mediate plant–plant interactions, including kin-recognition, priming and competition^18–20^. Moreover, plants are known to modify their VOC emissions in the presence of different neighbouring plants^21^ and even have ‘geographic dialects’^22^, responding strongly to cues of other plants from the same region.

Given the important ecological roles of VOCs in the context of plant invasion, it is relevant to explore, among other questions, if invasive plants affect the volatile emissions of native plant species, and whether VOCs emitted by invasive plants disrupt communication between native plants and insects. Another aspect that remains poorly explored in the literature is how biological control agents (insects), introduced to control invasive weeds, respond to volatiles from non-host native plants, despite increasing awareness that volatile cues are essential for host selection of these phytophagous insects^23,24^.

To address these knowledge gaps, we explored interactions between native and introduced plant and insect species that coexist on the North Island Central Plateau of New Zealand. This is a sub-alpine environment where the European introduced plants, *Cytisus scoparius* (Scotch broom; henceforth broom) and *Calluna vulgaris* (heather) are highly invasive. Information about broom’s introduction and establishment is scarce. Its potential to invade the area was only realised in the 1960s^25^ and although several biocontrol agents have been introduced in this region, they have only been partially successful^26^. Heather was deliberately introduced to the Central Plateau in 1912^27^ and is now widespread, contributing to a decline in plant and arthropod biodiversity^28–30^. To help control the spread of heather in this region, *Lochmaea suturalis* (heather beetle), imported from the United Kingdom, was released in 1996 and despite initial poor performance, it is now successfully controlling this plant in some areas^26,31^.

To determine the impact of invasive plants’ airborne cues on a native plant’s VOCs, we explored the volatile emissions of the native shrub, *Leptospermun scoparium* (mānuka), when grown adjacent to heather or broom in a semi-field experiment using potted plants without above- or below-ground physical contact. Mānuka is an economic and culturally relevant plant species in New Zealand, due to its use in premium honey production and being considered a ‘treasured’ (Taonga) species for indigenous Māori communities^32^. Mānuka above-ground volatile emissions have only recently been characterised^33,34^, and under field conditions, VOC emissions of mānuka were observed to be lower at sites where it co-occurs with invasive species, suggesting that invasive species could disrupt the communication networks of this native shrub^33^. However, the ability of this native plant to perceive and respond to invaders’ VOCs has not been previously investigated.

**Figure 1:**
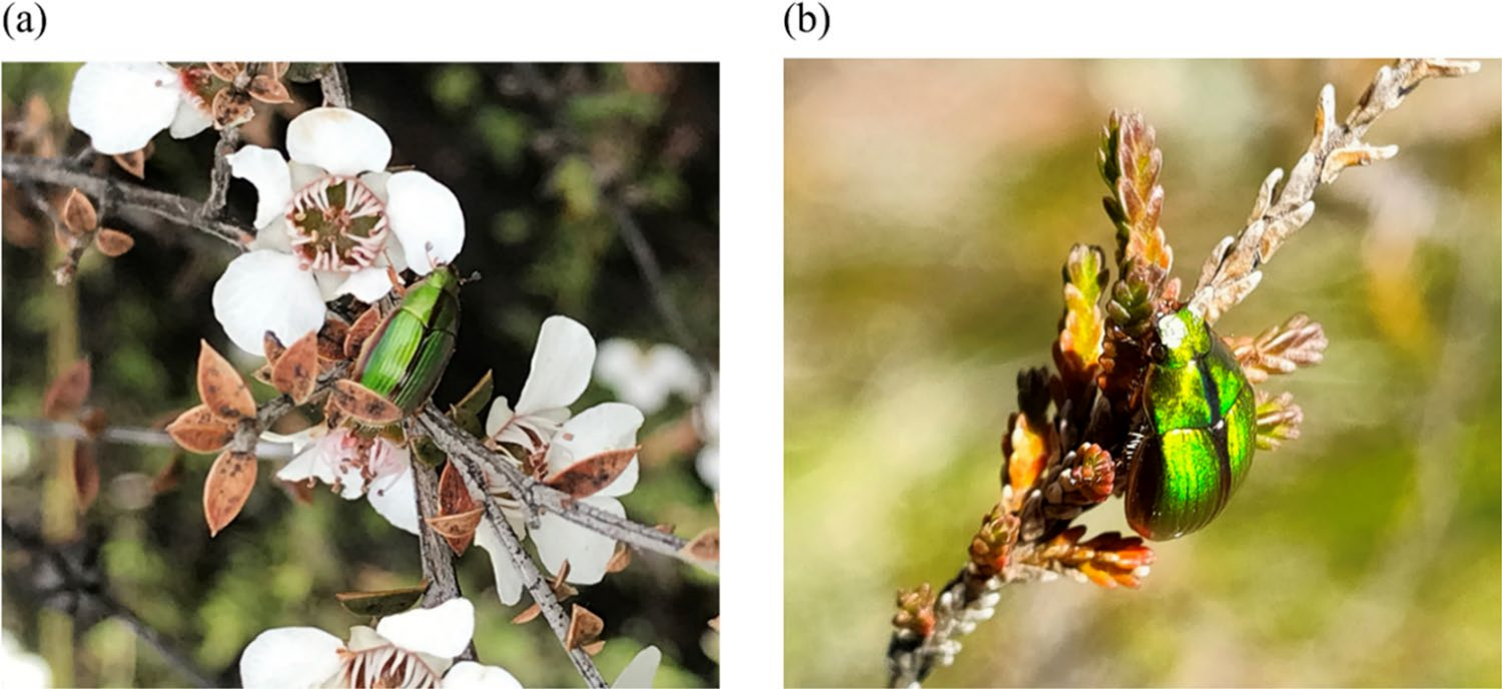
Photos of adult *P. festiva* on its: (**a**) host (mānuka) and (**b**) non-host (heather). Photographs were taken on the North Island Central Plateau, New Zealand. Credit: Evans Effah and Benjamin Pearson.

To explore potential disruption in native plant–insect communication by invasive plant species, we performed a series of laboratory assays to investigate the host-selection of the endemic mānuka beetle, *Pyronota festiva* when presented with volatiles from its host plant (mānuka) or heather individually, and the two plants simultaneously, and conducted follow up preference/feeding assays using plant foliage. In a previous field study, we recorded lower numbers of *P. festiva* on its preferred mānuka host in areas invaded by exotic weeds^33^, and we have on several occasions observed the beetle on heather in the field (personal observations; Fig. 1). However, whether *P. festiva* will feed on heather when mānuka is scarce remains unknown. Similar bioassays were conducted for the introduced biocontrol agent (the heather beetle, *L. suturalis*) to explore its behaviour towards a native plant and to confirm that it retains its host specificity.

## Materials and methods

All the methods, including plant and invertebrate collection, followed relevant institutional and national guidelines and legislation.

### Volatile emissions of mānuka neighbouring conspecifics or invasive species

Potted wild mānuka plants (≈ one year old) were purchased from TreesforBees Plant Nursery, New Zealand, in May 2019. These plants were transplanted into 3.3 L plastic pots with the following soil mix: 80% bark fines, 20% pumice, 6 kg Agroblend, 3 kg dolomite, 1 kg aglime and 1 kg gypsum per m^3^. Young heather and broom plants were collected from a wild population on the North Island’s Central Plateau, New Zealand, in August 2018 and transplanted into 3.3 L plastic pots, keeping the root-bound soil intact. All plants were maintained under the same conditions in an outdoor cage before commencement of experiments.

In September 2019, the potted plants were transferred to experimental plots (2 m × 3 m) in open pasture land (Long. 175.612167–Lat. − 40.387417) at Massey University. The plants were placed on weed matting and, using an automatic sprinkler system, watered twice and four times daily during spring and summer, respectively. The vegetation surrounding each plot was kept low by periodic scything. Each plot consisted of 10 mānuka plants intermingled with and an equal number of conspecifics or one of the two invaders (heather or broom), ensuring no above-ground or below-ground physical contact. Each treatment was replicated twice (×2), and all plots were set ≥ 6 m apart (Supplementary Fig. S1).

VOCs were collected from 6 healthy mānuka plants per plot from 7 to 9th January 2020. All volatile collections were done under sunny, dry conditions, and samples were collected from treatments simultaneously to account for collection time effects. Headspace volatiles of mānuka were collected using the “push–pull” sampling technique^35^. Briefly, proportions of mānuka foliage were bagged in new Glad ® oven bags. Carbon-filtered air was simultaneously pushed into and pulled out of bags through PTFE tubes connected to a portable volatile collection system pump (PVAS22; Volatile Assay Systems Rensselaer NY, USA). Compounds were trapped onto volatile collection filters containing 30 mg HayeSep Q adsorbent (Volatile Assay Systems Rensselaer, NY, USA). Each volatile collection lasted 2 h. The bagged foliage was then excised, oven-dried (60 °C for 72 h) and used to estimate VOC emission per dry weight (g).

Volatile collection filters were eluted using 200 μL of 99% hexane (Sigma Aldrich) containing 10 ng/mL nonyl acetate (C_11_H_22_O_2_) (Sigma Aldrich) as an internal standard. The eluted samples were analysed using gas chromatography coupled to mass spectrometry (GC–MS) (QP2010; GCMS Solution version 2.70, Shimadzu Corporations, Kyoto, Japan) with a 30 m × 250 μm × 0.25 μm TG-5MS column (Thermo Fisher Scientific, Waltham, MA, USA). The GC–MS programme followed^35^, and compounds were tentatively identified by comparing them to the National Institute of Standards library and confirmed using commercial standards when available. The air in clean oven bags without plants (blanks) was analysed, and contaminants were excluded from the analysis. Compounds identified from plants in both plots were pooled for respective treatments for analysis.

### Host-selection and feeding behaviours of adult *Pyronota festiva* (mānuka beetle) and *Lochmaea suturalis* (heather beetle)

#### Beetle and plant collection

Adult *Pyronota festiva* and *Lochmaea suturalis* were collected from the Central Plateau in early summer 2021 using a beating tray. The collected beetles were maintained in cages and fed with their host plant foliage under temperature-controlled conditions (20 °C) with a 12:12 h light/dark cycle. Young heather plants were collected from the Central Plateau, while mānuka plants (≈ 6 months old) were purchased from a commercial nursery. Both plant species were maintained in an outdoor cage for ≈ 7 months before the experiment. Each plant used for the bioassays was healthy and undamaged. Plants were removed from the outdoor cage once their foliage was excised for bioassays to avoid inducing changes in the volatile profiles of the remaining healthy plants. Beetles were starved 24 h prior to their trial, and each beetle was tested only once.

#### Y-tube bioassays

We tested the preference of adult *P. festiva* and *L. suturalis* using a glass Y-tube olfactometer. The Y-tube was laid horizontally without any inclination on a benchtop with a white background. Y-tube arms were 2 cm internal diameter × 13.2 cm long. Using a portable volatile collection pump (PVAS22; Volatile Assay Systems Rensselaer NY), connected with PTFE tubes, carbon-filtered air was pushed at a rate of 0.8 L min^−1^ into two separate 20 cm × 24 cm glass chambers. Each chamber contained a different treatment (i.e., clean air, heather, or mānuka foliage). For the plant treatments, 4 g of the respective plant foliage were placed in each chamber. Air from the chambers was then pushed into the assigned Y-tube arm. The trials were conducted in a temperature-controlled room (22 °C), with no overhead lighting but the Y-tube was illuminated by a centrally positioned 50-W incandescent lamp.

*Pyronota festiva* and *L. suturalis* were separately offered a choice between Y-tube arms with the following olfactory cues: (1) heather or clean air, (2) mānuka or clean air and (3) heather or mānuka. Each beetle was placed at the release point and given 10 min to respond to the treatment. A choice was recorded when a beetle moved past an arm’s halfway mark and stayed there for 30 s. Beetles that did not choose within the allocated time were noted as a no choice. Thirty (30) insects were tested for each treatment per beetle species, and the Y-tube was cleaned and rotated after each trial. Foliage in the glass chambers was replaced with fresh material after 10 trials, and the Y-tube system was thoroughly cleaned with non-scented soap and oven-dried for 30 min (80 °C) between treatments to prevent cross contamination.

#### Petri dish trials

Adult *P. festiva* and *L. suturalis*’ preference for their host and non-host plants was investigated in 9 cm × 1.5 cm Petri dishes lined with moistened filter paper. Twigs of healthy heather and mānuka plants inserted in separate water-filled Eppendorf tubes were used as the tested plant materials, while a green non-scented plastic was used as a blank. The following treatment combinations were tested for each beetle species: (1) heather + blank, (2) mānuka + blank and (3) heather + mānuka. One beetle was placed in the middle of a Petri dish containing one of the treatments, with 30 replicates conducted for each beetle species. The location of each beetle on either plant or blank was recorded at 0.25, 0.5, 1, 2, 16 and 32 h, after which the foliage was visually examined and recorded as damaged or undamaged. The trials were conducted in a temperature-controlled room (22 °C) with a 16:8 h light/dark cycle.

### Data analysis

All statistical analyses were performed using R version 4.1.0^36^. To assess the effect of invasive plants’ airborne cues on mānuka VOC emissions, we used linear discriminant analysis (LDA) to establish if we could classify mānuka into the the three neighbour-treatments (i.e., heather, broom or conspecifics) based on the individual volatile compounds. The data was standardised, and LDA was performed using the package “Mass”^37^. In addition, we counted the number of compounds that were abundant in the plants’ headspace and compared them between treatments using a generalised linear model (GLM) with Poisson distribution (log-link). The likelihood ratio test was used to estimate the significance of the predictor, and when significant, the “relevel” function was used to construct a series of pairwise comparisons. We also grouped the volatile compounds into their major chemical classes (Supplementary Table S1) and compared them between treatments using GLM, as already described, but with Gamma distribution (log-link). The beetle bioassay data were analysed using the two-tailed Chi-squared (*Χ*^2^) test.

## Results

### Volatile emissions of mānuka neighbouring conspecifics or invasive species

**Figure 2:**
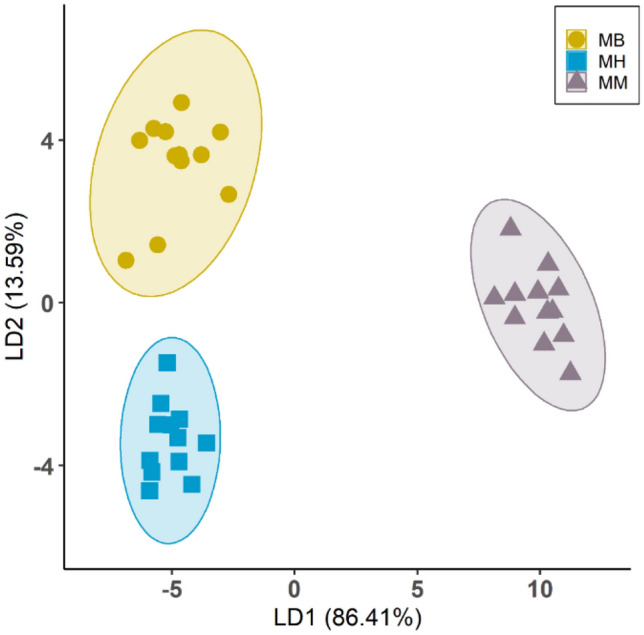
Linear discriminant analysis based on the aboveground volatile compounds identified from mānuka plants neighbouring broom (MB), heather (MH) or conspecifics (MM) (without above- or below-ground physical contact). Ellipses show a 95% confidence level.

Thirty-two volatile compounds, predominantly sesquiterpenes and monoterpenes, were identified as most abundant in the headspace of mānuka (Supplementary Tables S1 and S2). Based on these compounds, we used linear discriminant analysis (LDA) to classify mānuka into three distinct groups, with the results showing a clear separation between conspecific and heterospecific groupings (Fig. 2). Compounds including (*E*)-β-Caryophyllene, (*Z*)-β-Ocimene, (*Z,E*)-α-Farnesene, α-Amorphene, α-Selinene, β-Elemene, Calamenene, δ-Cadinene, Humulene, Isoledene, Limonene, Methyl salicylate and Nerol had higher loading scores, which correspond with the higher emission of these compounds by mānuka neighbouring conspecifics (Supplementary Tables S2 and S3).

**Figure 3:**
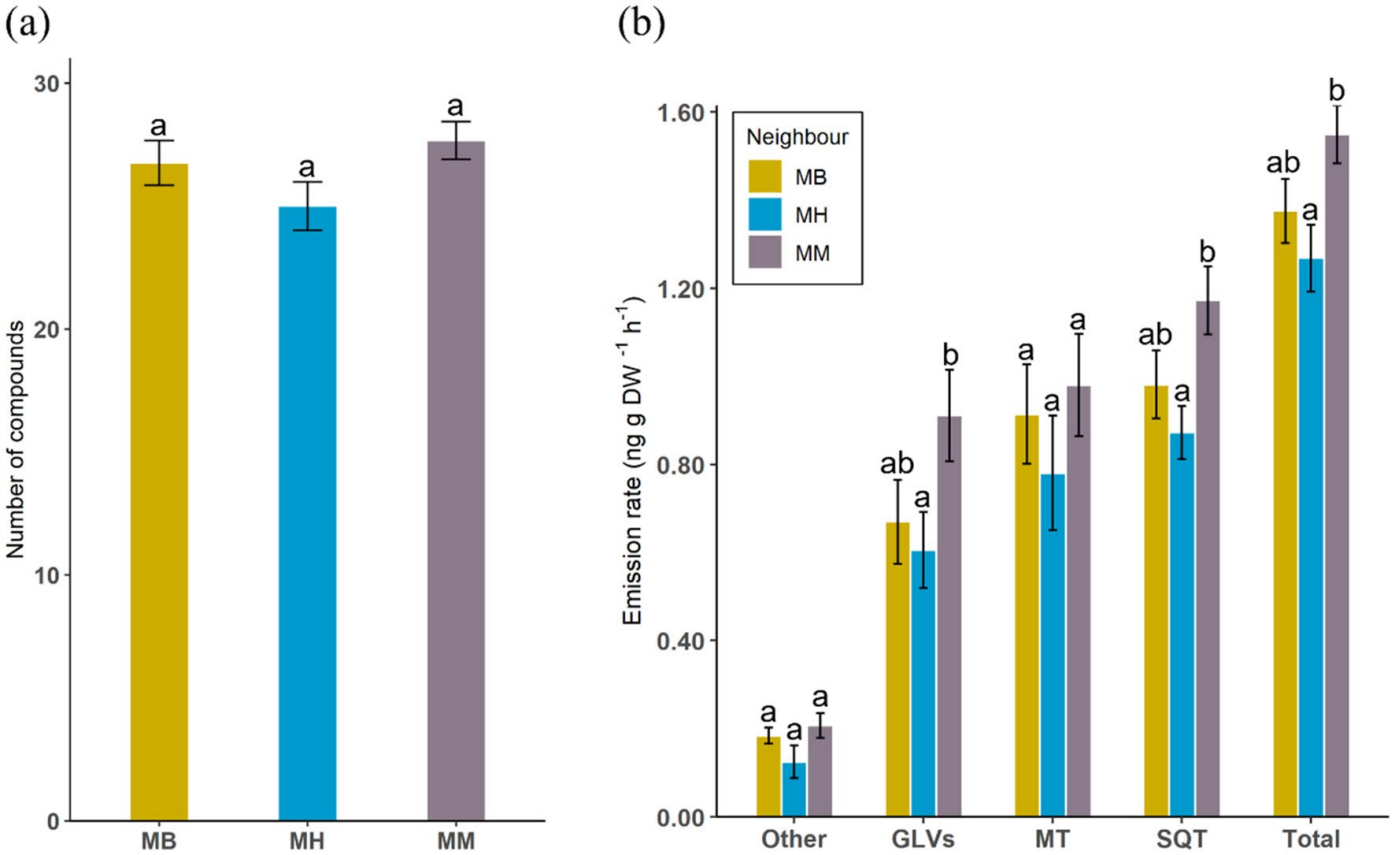
Comparison of (**a**) number of compounds and (**b**) emission rates of volatile compounds released by mānuka plants neighbouring either broom (MB), heather (MH) or conspecifics (MM). GLVs—green leaf volatiles, MT—monoterpenoids, SQT—sesquiterpenes, Other—other volatiles, Total—total emissions. Different letters indicate significant differences based on generali*s*ed linear models (*n* = 12).

The total number of compounds in the headspace of mānuka did not differ significantly between the three treatments (*X*^2^ = 1.67, df = 2, *P* = 0.433, Fig. 3a). Total emissions (*X*^2^ = 7.91, df = 2, *P* = 0.019, Fig. 3b) and those of specific chemical classes differed between treatments (Supplementary Table S4). Green leaf volatiles (*X*^2^ = 6.82, df = 2, *P* = 0.033) and sesquiterpenes (*X*^2^ = 10.93, df = 2, *P* = 0.004) were emitted in significantly higher amounts in the mānuka-mānuka treatment, while monoterpenoids (*X*^2^ = 0.73, df = 2, *P* = 0.698) and other volatiles (*X*^2^ = 2.79, df = 2, *P* = 0.248) did not show significant differences between treatments.

### Host-selection and feeding behaviours of adult *Pyronota festiva* (mānuka beetle) and *Lochmaea suturalis* (heather beetle)

**Figure 4:**
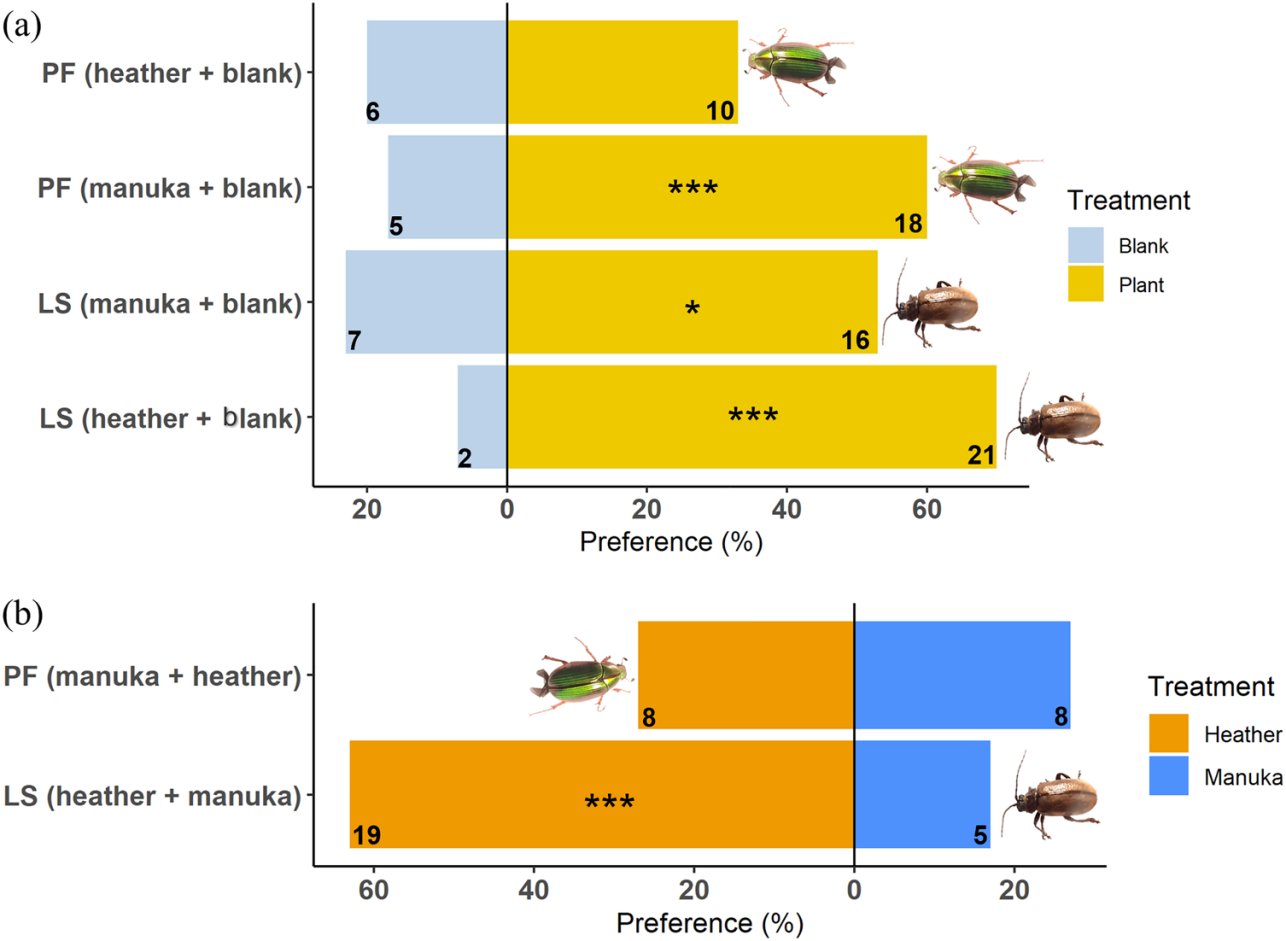
Adult *P. festiva* (PF) and *L. suturalis* (LS) choices in a Y-tube olfactometer (**a**) paired choice test offering host and non-host plants vs clean air (blank) and (**b**) paired choice test offering host vs non-host plants. X-axes show beetle preference (%), and y-axes show beetle species and the source of odours (plant or blank) in the Y-tube arms. E.g., PF (heather + blank) means *P. festiva* presented with heather plant volatiles vs blank, and so on. Numbers indicate frequencies (*n* = 30). All statistically significant differences between choices shown are based on χ^2^ test (*, *P* < 0.05; **, *P* < 0.01; ***, *P* < 0.001).

In the Y-tube olfactometer trials, when plants were paired with blank (clean air), *P. festiva* was significantly attracted to its host plant (*X*^2^ = 10.15, df = 1, *P* = 0.001) and had a slight, but not significant, preference for the invasive heather over clean air (*X*^2^ = 0.77, df = 1, *P* = 0.381, Fig. 4a). However, *P. festiva* could not differentiate between its host plant volatiles and those of heather in the paired choice test and selected equally the respective Y-tube arms (*X*^2^ = 0.00, df = 1, *P* = 1.000, Fig. 4b).

Adult *L. suturalis,* on the other hand, showed a significant preference for one of the treatments in all trials, where it preferred its host plant’s volatiles (*X*^2^ = 22.84, df = 1, *P* < 0.001), and those of mānuka (*X*^2^ = 4.51, df = 1, *P* = 0.034) compared to clean air (Fig. 4a). But, unlike *P. festiva, L. suturalis* preferred the volatiles of its host plant when offered the two plants simultaneously (*X*^2^ = 11.74, df = 1, *P* = 0.001, Fig. 4b).

**Figure 5:**
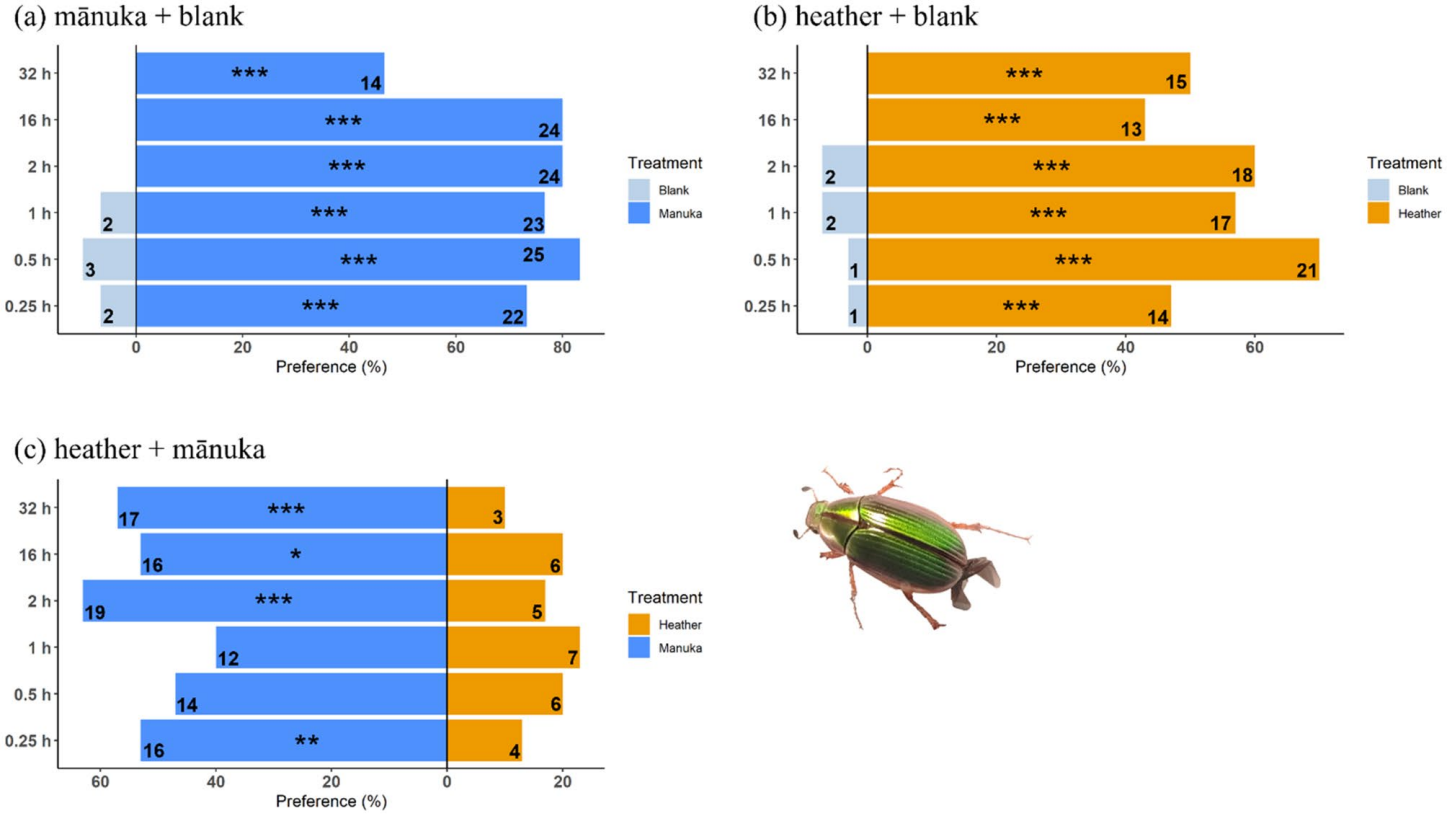
*P. festiva* host selection recorded at different times in a Petri dish. Numbers indicate frequencies (*n* = 30). All statistically significant differences between choices shown are based on χ^2^ test (*, *P* < 0.05; **, *P* < 0.01; ***, *P* < 0.001).

Beetle host selection and feeding preferences for their host and non-host plants were also assessed in Petri dishes for 32 h, with observations at 0.5, 1, 2, 16 and 32 h. At any measured time, *P. festiva* showed a significant preference for mānuka and heather cues over a blank (Fig. 5a,b). When offered mānuka and heather cues simultaneously, *P. festiva* showed a stronger preference for its host plant, although heather attracted some individuals (Fig. 5c, Supplementary Table S5).

**Figure 6:**
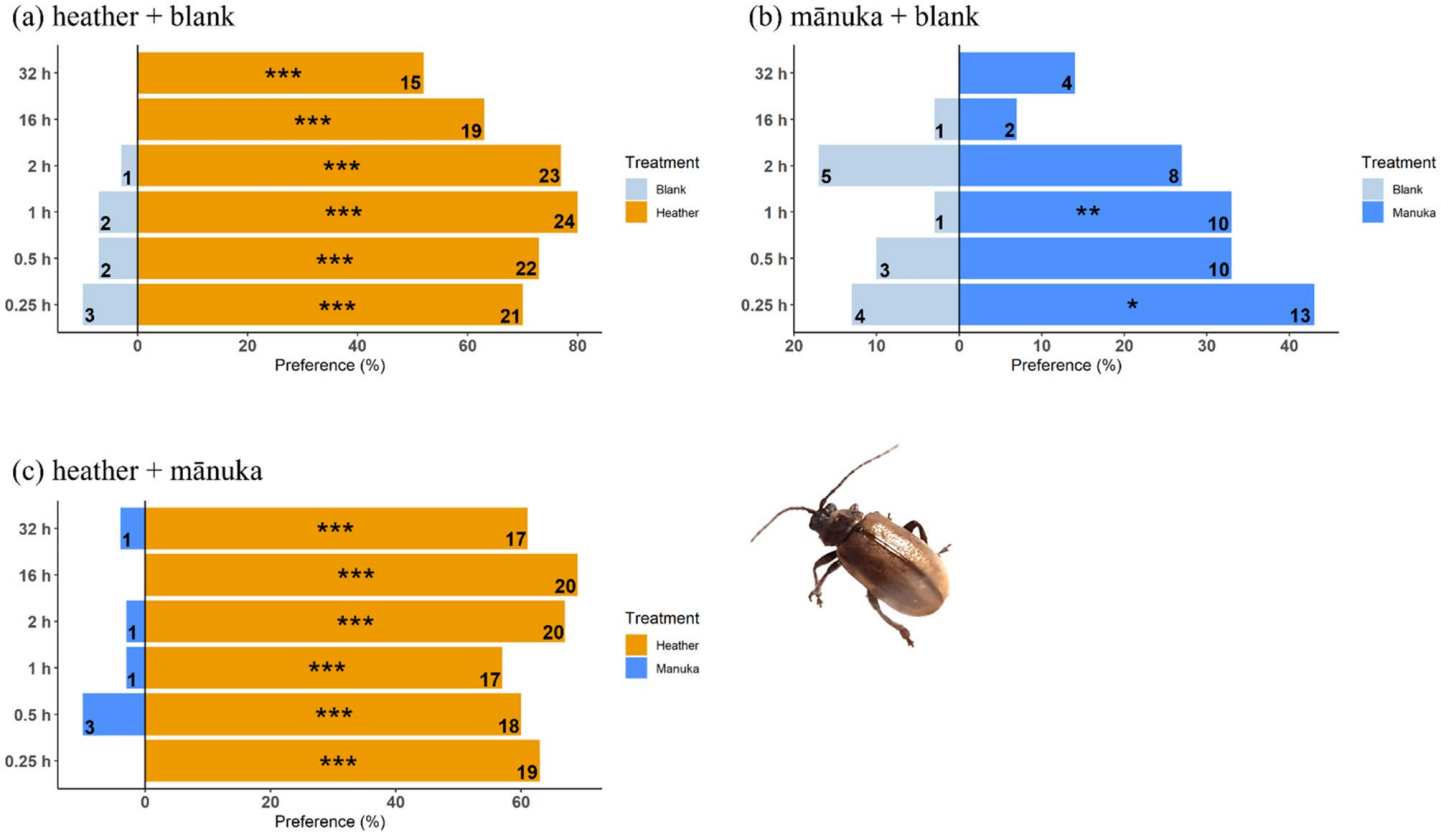
*L. suturalis* host selection recorded at different times in a Petri dish. Numbers indicate frequencies (*n* = 30). All statistically significant differences between choices shown are based on χ^2^ test (*, *P* < 0.05; **, *P* < 0.01; ***, *P* < 0.001).

Similarly, *L. suturalis* showed a significant preference for its host plant at all measured times and sometimes for mānuka when heather was not available (Fig. 6a,b). The beetle selected its host plant over mānuka when presented with the two simultaneously (Fig. 6c, Supplementary Table S5).

**Figure 7:**
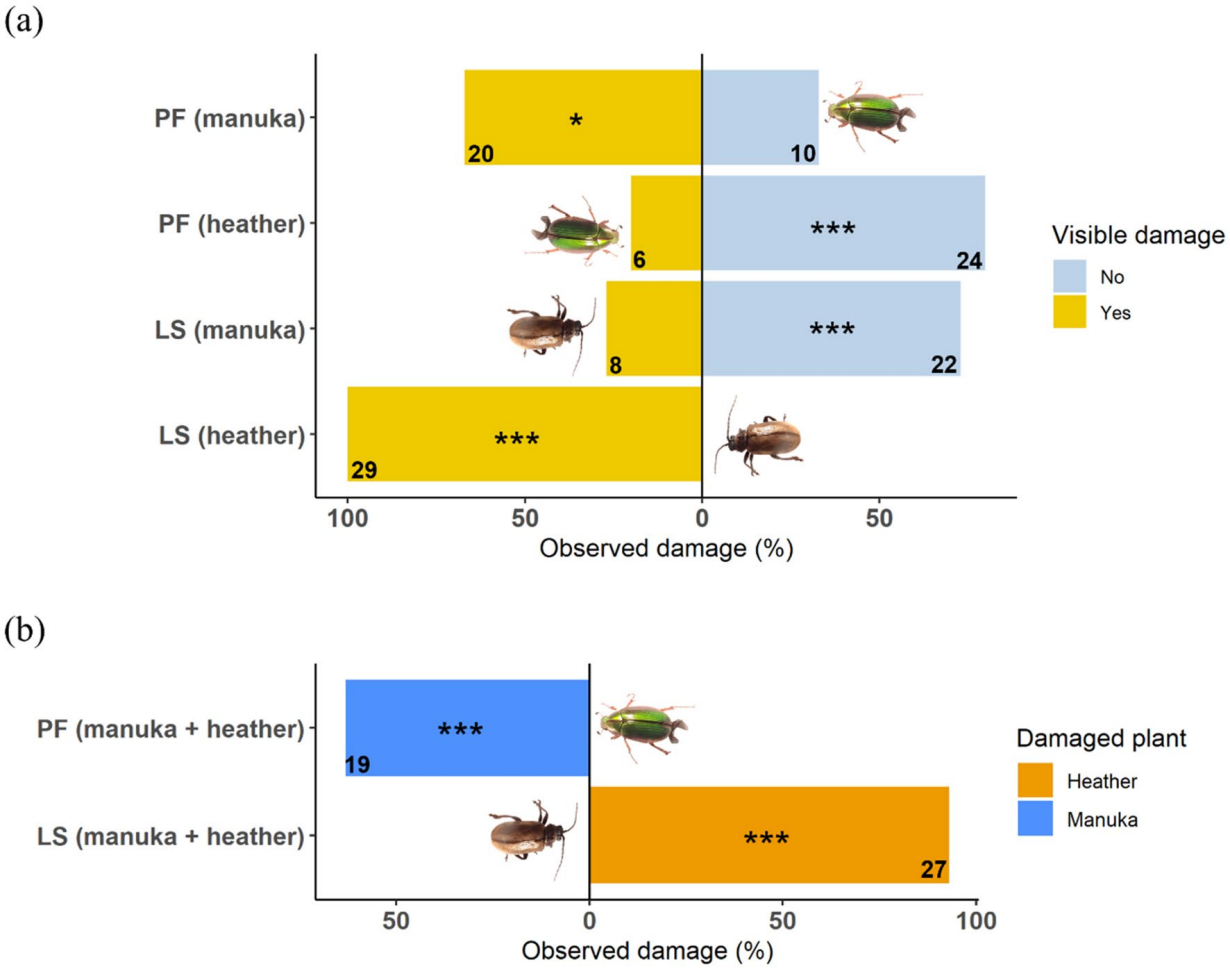
Observed feeding damage caused by *P. festiva* and *L. suturalis* when (**a**) only one plant was offered (either host or non host) vs a blank or (**b**) a paired choice between host and non-host plant was offered. (**a**) “Yes” and “No” means damaged and undamaged, respectively. X-axes show observed foliar damage (%), and y-axes show beetle species and treatment. E.g., PF (mānuka) means *P. festiva* offered with mānuka, and so on. Numbers indicate frequencies (*n* = 30). All statistically significant differences between choices shown are based on χ^2^ test (*, *P* < 0.05; **, *P* < 0.01; ***, *P* < 0.001).

After 32 h, beetles were removed from the Petri dishes, and foliar feeding damage was visually inspected. We found significant damage when *P. festiva* (*X*^2^ = 5.40, *P* = 0.020) and *L*. *suturalis* (*X*^2^ = 54.07, *P* < 0.001) were only offered their respective host plants (Fig. 7a). However, when offered only their non-host plants, damage signs were extremely low on heather offered to *P. festiva* (*X*^2^ = 19.27, *P* < 0.001) and on mānuka offered to *L*. *suturalis* (*X*^2^ = 11.27, *P* = 0.001), (Fig. 7a). About 60% of *P. festiva* (*X*^2^ = 24.96, *P* < 0.001) and 90% of *L*. *suturalis* (*X*^2^ = 46.84, *P* < 0.001) fed on their respective host when offered simultaneously with a non-host plant, with none of the beetles feeding on the non-host plant (Fig. 7b).

## Discussion

There is a vast body of literature exploring the ecological roles of plant volatiles and allelopathic potential of invasive plants—focused mainly on root exudates (see excellent reviews by^6,38–40^) but comparatively few studies have explored the role of volatile organic compounds (VOCs) in interactions between native and invasive species. In this study, we investigated some interactions between native and introduced plants and insects mediated by VOCs, including (1) the effect of invasive plants on a native plant’s VOC emissions, and (2) the host-selection and feeding preference of a native insect and an introduced biocontrol agent when presented with volatiles only and a combination of cues from host and non-host plants.

### Impacts of neighbouring plant identity on native plants’ VOC emissions

The notion of ‘talking plants’ has been around the scientific literature for about fifty years, since Rhoades first reported that uninfested Sitka willow (*Salix sitchensis*) in close proximity to herbivore-infested conspecifics expressed higher levels of herbivore resistance than plants of the same species growing further away^41,42^. Since then, this phenomenon has been reported for multiple species^43^, and it is now widely accepted that healthy plants or plant parts can detect herbivore-induced volatiles from a neighbour (or attacked plant part) and initiate changes in their defensive chemistry to prepare for future attack (e.g., through priming). Work by Barbosa et al.^44^ highlighted that plant associations could increase (associational susceptibility) or decrease (associational resistance) the likelihood of plant detection by herbivores, suggesting volatiles might play a role. Further to this, several reports (e.g.,^21,45^) showed that the volatile compounds emitted by a plant depended on the neighbouring species and that responses would depend on whether the plant is surrounded by kin or non-kin. Karban and co-workers described the occurrence of ‘geographic dialects’, with plants from different ranges having distinct volatile profiles and responding strongly to VOCs emitted by others in the same geographical area^22^. This evidence invites the question if native plants can detect and respond to the VOCs of invasive plants.

Here, in a semi-field experiment, we explored the VOC emissions of the New Zealand native plant, mānuka, in the presence of two exotic invasive species, heather and broom (without physical above- or below-ground contact). The results reveal significantly lower VOC emissions by mānuka neighouring invasives than conspecifics, particularly when paired with heather, supporting previous field observations where mānuka was observed to have lower VOC emissions in invaded sites^33^, and suggest that above-ground volatiles alone are at least partly accountable for this response.

Increasingly, reports show that the species composition of neighbouring vegetation strongly influences VOC emissions. For instance, *Trifolium pratense* reduced its volatile emissions when growing with conspecifics, possibly to avoid herbivore attraction and reduce nearby heterospecifics’ ability to eavesdrop on herbivore information shared between conspecifics^21,45^. Contrary, lower emissions have been reported for *Pinus halepensis*^46^*, Rosmarinus officinalis,* and *Cistus albidus*^47^ under interspecific interactions, showing that responses to neighbouring plants may vary depending on the species involved.

We hypothesise that changes in VOC emissions by native plants can occur via two non-exclusive mechanisms (a) as a direct response to the cues of a competing plant, e.g., volatile and non-volatile compounds or (b) as an indirect response to environmental changes caused by invasive species, e.g., changes in light or soil nutrients. The contribution of these direct and indirect factors is difficult to disentangle under field conditions, but previous studies^21,45^ show that aboveground VOCs alone are sufficient to elicit changes in the emissions of receiving plants and that responses will vary depending on the neighbour’s identity. We also acknowledge the possibility of ‘chemical camouflage’ (i.e., the adsorbance and re-release of neighbouring plants’ chemical compounds) as a potential factor that could contribute to higher emissions in some plots^48–50^. Therefore, further studies are required to elucidate the mechanisms behind the observed phenomenon.

The exact mechanism of plant “olfaction” (i.e., perception of volatile cues by a plant) is still not well-understood. Questions like whether plants have VOC-sensing receptors and other transporters or VOCs are perceived through direct modification of cell membranes, remain unanswered^51,52^. Nevertheless, receiving plants use volatile cues to establish their neighbours’ identity and state, informing their decisions about imminent threats such as competition and herbivory^19,20,39,53^. Therefore, upon deciphering neighbours’ volatile cues, it is plausible that plants alter their emissions for a number of purposes (a) to benefit conspecifics (e.g., increased emission to attract pollinator and herbivores’ natural enemies), (b) to harm competitors (e.g., increased emission for VOC-mediated allelopathy), (c) to reduce plant apparency to antagonists (i.e., reduced emission to avoid herbivores), or (d) as preparedness for competition (e.g., reduce emissions to reallocate resources to growth and reproduction).

In the case of mānuka, the reduced emissions when exposed to the cues of aggressive neighbours could be preparedness for competition^39,54^. Thus, the plant lowers its emission to reallocate much-needed resources to compete with the invaders, since VOC production comes at a cost^55^. Alternatively (or simultaneously) reduced emissions in response to an invader’s cues could be a means to minimise apparency to avoid nectar robbers or herbivores that can negatively affect its fitness^44^. Testing these hypotheses would be another step toward understanding the impact of invasive species on natives’ chemical communication in this and similar systems.

### Herbivores’ response to host and non-host cues

Plant volatiles play a vital role in host plant selection by phytophagous insects^16^. Most insects appear to distinguish host and non-host plants based on specific blends of ubiquitous volatiles, although some specialists are known to use taxonomically restricted compounds (such as isothiocyanates in cruciferous plants) to find their hosts^16^. However, responses to plant volatiles are not entirely fixed, since early feeding experience and learning can play a role in determining future choices^56–59^, allowing for some behavioural plasticity. During plant invasions, native insects are confronted with new olfactory cues from plants they did not co-evolve with. Likewise, introduced biocontrol agents will experience a similar challenge when faced with native plants. Exploring if these new cues affect native insects’ host-selection process is essential to understanding the ecological impacts of invasive plants (i.e., potential disruption of native plant–insect communication), and to ensure the safety of introduced biocontrol agents.

In this work, we explored the behavioural responses of the native mānuka beetle (*P. festiva*) and the introduced heather beetle (*L. suturalis*) towards volatiles of their host and non-host plants and their combination in a Y tube olfactometer. Our results showed a strong attraction response by the native mānuka beetle towards its host-plant’s volatile cues, when plant volatiles were presented against clean air, but no preference when host and non-host cues were presented simultaneously. In contrast, the introduced heather biocontrol agent showed an interest in the non-host plant (mānuka) when presented against clean air, but showed a much stronger preference for its host’s volatile cues irrespective of when presented alone or in combination with non-host cues.

Host-searching and selection by adult phytophagous insects involves complex decisions like prioritising their own diet versus choosing plants that would be best for their offspring^16,60^. This may be a challenge for species like *P. festiva* where adults and juveniles feed on different plants or organs*,* with grubs feeding on roots of different species, whereas adults feed mainly on the foliage of mānuka plants^61–63^.

The host range for *P. festiva* includes pasture, *Leptospermum scoparium* (mānuka), *Kunzea ericoides* (kānuka), *Discaria toumatou* (matagouri) and even invasive *Rosa rubiginosa* (briar)^61–63^. Considering this, the beetle is probably either attracted by ‘signatory compounds’ shared by common hosts or simply avoids cues from non-hosts^64^. Analyses of the volatile profiles of *P. festiva*’s host (mānuka) and non-host plant (heather) reveal that they share many compounds, while some other compounds differ between species^33,35^. Further studies involving electroantennography could be useful in identifying the compounds (attractants or deterrents) that are relevant in the host selection of *P. festiva*.

We found that *P. festiva* was not significantly attracted to heather’s volatile cues when offered in the no-choice test but was equally attracted to mānuka and heather cues when provided simultaneously in the Y-tube, suggesting that the presence of heather may interfere with the beetle's host searching behaviour. However, in the Petri dish trial, where other cues (i.e., visual, gustatory, and tactile) were present, *P. festiva* did not feed on heather when offered simultaneously with mānuka in the Petri dish, suggesting that visual, gustatory or tactile cues play an important role in host acceptance for this species.

*Lochmaea suturalis,* on the other hand, is a monophagous insect and was selected as a biocontrol agent for heather on the Central Plateau because of the high levels of damage it causes to its host plant in Europe^65^. *L. suturalis* was first released on the Central Plateau in 1996, following years of host-range testing but initially established poorly, attributed to adverse weather conditions^31^ and possibly low foliar nitrogen levels^66^ and the consequences of genetic bottlenecking^67^. Subsequent releases have been more successful, with beetle outbreaks causing significant damage to heather in many areas^26^. Recent evidence also shows that heather produces many volatile compounds, including green leaf volatiles, terpenes and aldehydes^35,68^, which may be crucial in communicating with its natural enemy, *L. suturalis.*

Our laboratory assays show that *L. suturalis* is significantly attracted to its host-plant volatile cues when offered alone or simultaneously with that of a non-host plant in the Y-tube. Nevertheless, the beetle was significantly attracted to non-host volatile cues when presented against a blank, raising questions about its foraging behaviour in areas with low heather densities. The Petri dish trials somewhat answered this. The beetle chose its host plant exclusively over mānuka when both were offered simultaneously. Although the beetle selected mānuka when given no other choice, this was not often significant, and only a few (26.7%) of the tested beetles attempted to feed on mānuka in the absence of its host plant.

Host-switching or host-range expansion by biocontrol agents is rare in contemporary biocontrol programmes partly because of sufficient pre-release tests to ensure high host specificity, but it may sometimes occur^69^. For instance, a study in Nebraska, USA, showed that the introduced biocontrol agent *Rhinocyllus conicus* attacks native *Cirsium undulatum* significantly more in landscapes invaded by the exotic *Carduus nutans* than in agriculture landscapes and other areas without *Carduus nutans*, highlighting the risk of native plants serving as secondary hosts^70^. In our trials, we did not find substantial evidence of *L. suturalis* feeding on mānuka, suggesting that it retains its high host-specificity, and that host switch or host range expansion is unlikely to occur.

Since both beetle species were collected in the field as adults and non-sexed, we cannot provide further detail regarding their previous feeding experience or whether sexes differ in their behaviour and preferences. We, therefore, encourage future studies to explore these interactions using laboratory-reared beetles and test separately for larvae, adults, and different sexes.

## Conclusion

Our results demonstrate that invasive plants can influence native plants’ volatile emissions. In this case, we found reduced VOC emissions in a native plant (*Leptospermum scoparium*) neighbouring the invasive weed *Calluna vulgaris*. Alterations in VOC emission could be the result of responses to environmental changes induced by invaders or to their chemical cues. Alternatively, native plants could passively adsorb and re-release neighbouring plants’ VOCs. Regardless of the mechanism, changes in native plants’ volatile profiles could interfere with their chemical communication and interactions. Our data also show that native insects’ chemical interactions with their host can be disrupted by invasive plants. We found that a native insect (*Pyronota festiva*) was not successful in discriminating between its host plant and an invasive non-host when their volatiles were presented simultaneously, suggesting that native insects may face challenges finding their host in invasive plant-dominated landscapes. However, the native insect showed a clear preference for its host plant in feeding assays where other cues were present, highlighting the importance of non-volatile cues. Our results also reinforce that the introduced biocontrol agent against heather (*Lochmaea suturalis*) is highly host-specific and does not pose any serious threat to mānuka and possibly other non-target plants. Together, these results contribute to filling the knowledge gap on the role of plant volatiles in interactions between native and introduced species, however, multiple questions remain open for future exploration.

## Supplementary Information

Supplementary Information.

## Data Availability

All relevant data supporting the findings of this study are available from the corresponding author upon reasonable request.
