# Supplementary material for: Exploring plant volatile-mediated interactions between native and introduced plants and insects
Source: Sci Rep. 2022 Sep 14;12:15450. doi: 10.1038/s41598-022-18479-z (PMC9474884; doi:10.1038/s41598-022-18479-z)
Supplement: Supplementary file 1 — Supplementary Information. [file 41598_2022_18479_MOESM1_ESM.pdf]

## Supplementary material

**Figure S1.** Experimental design for mānuka volatile collection. (a) mānuka in conspecific stand, (b) mānuka paired with heather and (c) mānuka paired with broom. Each plot was replicated twice.

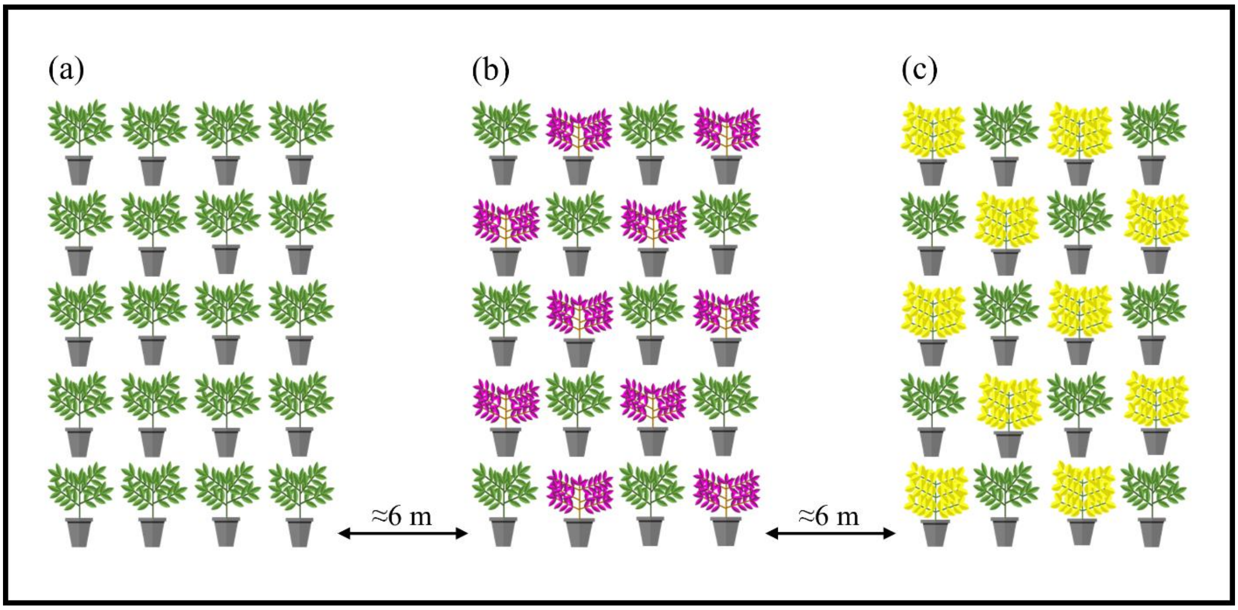

**Table S1.** Mean emission rate of volatile compounds identified from mānuka at individual plots. Each treatment was replicated twice. E.g., MB (plot 1) and MB (plot 2) were the two plots where manuka was paired with broom.

| | Mean ± SE emission rate per plot (ng $gDW^{-1}h^{-1}$) | | | | | |
|---|---|---|---|---|---|---|
| **Compound** | **MB (Plot 1)** | **MB (Plot 2)** | **MH (Plot 1)** | **MH (Plot 2)** | **MM (Plot 1)** | **MM (Plot 2)** |
| **Green leaf volatiles** | | | | | | |
| (Z)-3-Hexenol [i] | 0.13 ± 0.06 | 0.31 ± 0.09 | 0.17 ± 0.07 | 0.26 ± 0.03 | 0.18 ± 0.08 | 0.49 ± 0.06 |
| (Z)-3-Hexenyl acetate [i] | 3.93 ± 1.83 | 5.49 ± 1.23 | 4.04 ± 2.02 | 3.73 ± 1.27 | 4.69 ± 1.39 | 14.35 ± 3.91 |
| **Monoterpenoids** | | | | | | |
| (*Z*)-β-Ocimene | 0.15 ± 0.07 | 0.24 ± 0.13 | 0.31 ± 0.11 | 0.13 ± 0.03 | 0.57 ± 0.31 | 0.29 ± 0.15 |
| β-Myrcene [i] | 1.63 ± 1.28 | 0.89 ± 0.43 | 0.32 ± 0.07 | 1.34 ± 0.93 | 0.47 ± 0.14 | 2.25 ± 1.87 |
| α-Pinene [i] | 2.61 ± 1.92 | 8.68 ± 3.30 | 8.06 ± 4.81 | 3.84 ± 1.68 | 6.24 ± 2.29 | 7.05 ± 3.87 |
| β-Pinene [i] | 0.26 ± 0.20 | 0.82 ± 0.28 | 0.72 ± 0.50 | 0.52 ± 0.26 | 0.71 ± 0.41 | 1.40 ± 0.73 |
| Sabinene | 0.09 ± 0.05 | 0.24 ± 0.10 | 0.11 ± 0.06 | 0.05 ± 0.03 | 0.14 ± 0.05 | 0.06 ± 0.04 |
| Nerol [i] | 0.67 ± 0.22 | 1.01 ± 0.25 | 0.80 ± 0.25 | 0.48 ± 0.15 | 1.85 ± 0.81 | 1.33 ± 0.48 |
| Limonene [i] | 0.22 ± 0.09 | 0.39 ± 0.11 | 0.24 ± 0.17 | 0.21 ± 0.05 | 0.43 ± 0.10 | 0.37 ± 0.14 |
| γ-Terpinene [i] | 0.15 ± 0.05 | 0.47 ± 0.14 | 0.06 ± 0.03 | 0.23 ± 0.09 | 0.44 ± 0.27 | 0.18 ± 0.05 |
| Eucalyptol [i] | 0.26 ± 0.25 | 1.91 ± 0.79 | 0.18 ± 0.13 | 0.38 ± 0.14 | 0.62 ± 0.27 | 0.40 ± 0.22 |
| α-Phellandrene [i] | 0.05 ± 0.04 | 0.19 ± 0.04 | 0.07 ± 0.04 | 0.09 ± 0.03 | 0.14 ± 0.05 | 0.11 ± 0.07 |
| **Sesquiterpenes** | | | | | | |
| (*E*)-α-Bergamotene | 0.19 ± 0.07 | 0.20 ± 0.03 | 0.22 ± 0.19 | 0.11 ± 0.05 | 0.14 ± 0.10 | 0.19 ± 0.08 |
| (*E*)-β-Caryophyllene [i] | 1.93 ± 0.66 | 5.39 ± 1.58 | 1.50 ± 0.40 | 4.87 ± 1.11 | 6.66 ± 3.51 | 5.63 ± 1.38 |
| (*Z,E*)-α-Farnesene | 0.09 ± 0.04 | 0.50 ± 0.17 | 0.10 ± 0.05 | 0.25 ± 0.15 | 0.60 ± 0.36 | 0.49± 0.16 |
| α-Amorphene | 0.19 ± 0.12 | 0.17 ± 0.10 | 0.05 ± 0.02 | 0.08 ± 0.03 | 0.16 ± 0.11 | 0.31 ± 0.25 |
| α-Cubebene | 0.54 ± 0.25 | 0.41 ± 0.10 | 0.14 ± 0.06 | 0.36 ± 0.11 | 0.58 ± 0.08 | 0.67 ± 0.29 |
| α-Gurjunene | 0.08 ± 0.04 | 0.09 ± 0.02 | 0.02 ± 0.01 | 0.07 ± 0.03 | 0.07 ± 0.03 | 0.09 ± 0.03 |
| α-Selinene | 0.06 ± 0.02 | 0.16 ± 0.02 | 0.05 ± 0.02 | 0.11 ± 0.03 | 0.14 ± 0.02 | 0.17 ± 0.10 |
| Aromadendrene | 0.07 ± 0.04 | 0.21 ± 0.08 | 0.10 ± 0.04 | 0.19 ± 0.07 | 0.10 ± 0.03 | 0.13 ± 0.08 |
| β-Elemene | 2.21 ± 1.68 | 1.38 ± 0.53 | 0.36 ± 0.15 | 0.84 ± 0.25 | 1.58 ± 0.74 | 3.37 ± 1.96 |
| Cadinadiene-1,4 | 0.25 ± 0.10 | 0.19 ± 0.04 | 0.06 ± 0.02 | 0.15 ± 0.01 | 0.24 ± 0.04 | 0.33 ± 0.11 |
| Calamenene | 0.83 ± 0.32 | 0.67 ± 0.13 | 0.25 ± 0.11 | 0.57 ± 0.07 | 1.37 ± 0.27 | 1.20 ± 0.39 |
| Chamigrene | 1.35 ± 0.57 | 0.68 ± 0.43 | 1.04 ± 0.32 | 0.84 ± 0.29 | 1.23 ± 0.48 | 3.62 ± 1.59 |
| Copaene | 0.12 ± 0.04 | 0.13 ± 0.02 | 0.03 ± 0.02 | 0.12 ± 0.01 | 0.12 ± 0.04 | 0.15 ± 0.04 |
| δ-Cadinene | 0.09 ± 0.04 | 0.11 ± 0.05 | 0.04 ± 0.02 | 0.05 ± 0.03 | 0.10 ± 0.02 | 0.36 ± 0.23 |
| Eudesma-4(14),11-diene | 0.70 ± 0.33 | 0.83 ± 0.21 | 0.82 ± 0.28 | 0.54 ± 0.18 | 0.73 ± 0.24 | 1.78 ± 0.55 |
| Humulene [i] | 0.14 ± 0.07 | 0.30 ± 0.09 | 0.09 ± 0.01 | 0.24 ± 0.04 | 0.30 ± 0.13 | 0.30 ± 0.07 |
| Isoledene | 0.46 ± 0.32 | 0.12 ± 0.07 | 0.03 ± 0.02 | 0.08 ± 0.04 | 0.25 ± 0.06 | 0.61 ± 0.39 |
| **Other volatiles** | | | | | | |
| Methyl salicylate | 0.25 ± 0.07 | 0.13 ± 0.07 | 0.34 ± 0.30 | 0.04 ± 0.02 | 0.33 ± 0.04 | 0.15 ± 0.03 |
| Nonanal [i] | 0.19 ± 0.02 | 0.17 ± 0.03 | 0.15 ± 0.03 | 0.13 ± 0.01 | 0.26 ± 0.03 | 0.14 ± 0.03 |
| 3-Methyl-1-butanol acetate | 0.12 ± 0.09 | 0.23 ± 0.09 | 0.08 ± 0.04 | 0.06 ± 0.03 | 0.19 ± 0.10 | 0.23 ± 0.11 |

[i] Compounds verified by authentic standards

**Table S2.** Mean emission rates for the pooled data. Plots with the same treatment in Table S1 were pooled to represent one treatment.

| | **Mean ± SE emission (ng gDW$^{-1}$h$^{-1}$)** | | |
|---|---|---|---|
| **Compound** | MB | MH | MM |
| **Green leaf volatiles** | | | |
| (Z)-3-Hexenol | 0.22 ± 0.06 | 0.21 ± 0.04 | 0.33 ± 0.07 |
| (Z)-3-Hexenyl acetate | 4.17 ± 1.08 | 3.89 ± 1.14 | 9.52 ± 2.45 |
| **Monoterpenoids** | | | |
| (*Z*)-β-Ocimene | 0.20 ± 0.07 | 0.22 ± 0.06 | 0.43 ± 0.17 |
| β-Myrcene | 1.26 ± 0.65 | 0.83 ± 0.47 | 1.49 ± 0.95 |
| α-Pinene | 5.64 ± 2.04 | 5.95 ± 2.51 | 6.64 ± 2.15 |
| β-Pinene | 0.54 ± 0.18 | 0.62 ± 0.27 | 1.06 ± 0.41 |
| Sabinene | 0.16 ± 0.06 | 0.08 ± 0.03 | 0.10 ± 0.03 |
| Nerol | 0.84 ± 0.17 | 0.64 ± 0.15 | 1.59 ± 0.46 |
| Limonene | 0.30 ± 0.07 | 0.23 ± 0.08 | 0.40 ± 0.08 |
| γ-Terpinene | 0.31 ± 0.09 | 0.14 ± 0.05 | 0.31 ± 0.14 |
| Eucalyptol | 1.08 ± 0.46 | 0.28 ± 0.09 | 0.51 ± 0.17 |
| α-Phellandrene | 0.12 ± 0.03 | 0.08 ± 0.02 | 0.12 ± 0.04 |
| **Sesquiterpenes** | | | |
| (*E*)-α-Bergamotene | 0.19 ± 0.04 | 0.16 ± 0.09 | 0.17 ± 0.06 |
| (*E*)-β-Caryophyllene | 3.66 ± 0.97 | 3.19 ± 0.76 | 6.15 ± 1.80 |
| (*Z,E*)-α-Farnesene | 0.30 ± 0.10 | 0.18 ± 0.08 | 0.54 ± 0.19 |
| α-Amorphene | 0.18 ± 0.07 | 0.07 ± 0.02 | 0.23 ± 0.13 |
| α-Cubebene | 0.47 ± 0.13 | 0.25 ± 0.07 | 0.63 ± 0.14 |
| α-Gurjunene | 0.08 ± 0.02 | 0.05 ± 0.02 | 0.08 ± 0.02 |
| α-Selinene | 0.11 ± 0.02 | 0.08 ± 0.02 | 0.16 ± 0.05 |
| Aromadendrene | 0.14 ± 0.05 | 0.15 ± 0.04 | 0.11 ± 0.04 |
| β-Elemene | 1.80 ± 0.85 | 0.60 ± 0.16 | 2.47 ± 1.04 |
| Cadinadiene-1,4 | 0.22 ± 0.05 | 0.11 ± 0.02 | 0.29 ± 0.06 |
| Calamenene | 0.75 ± 0.17 | 0.41 ± 0.08 | 1.28 ± 0.23 |
| Chamigrene | 1.02 ± 0.36 | 0.94 ± 0.21 | 2.43 ± 0.87 |
| Copaene | 0.13 ± 0.02 | 0.08 ± 0.02 | 0.13 ± 0.03 |
| δ-Cadinene | 0.10 ± 0.03 | 0.05 ± 0.02 | 0.23 ± 0.12 |
| Eudesma-4(14),11-diene | 0.76 ± 0.19 | 0.68 ± 0.17 | 1.25 ± 0.33 |
| Humulene | 0.22 ± 0.06 | 0.16 ± 0.03 | 0.30 ± 0.07 |
| Isoledene | 0.29 ± 0.17 | 0.05 ± 0.02 | 0.43 ± 0.19 |
| **Other volatiles** | | | |
| Methyl salicylate | 0.19 ± 0.05 | 0.19 ± 0.15 | 0.24 ± 0.04 |
| Nonanal | 0.18 ± 0.02 | 0.14 ± 0.02 | 0.20 ± 0.03 |
| 3-Methyl-1-butanol acetate | 0.18 ± 0.06 | 0.07 ± 0.02 | 0.21 ± 0.07 |

**Table S3**. Linear discriminant analysis loading scores for variables (volatile compounds) emitted by mānuka paired with different neighbors.

| Compound | LD1 | LD2 |
|---|---|---|
| (*E*)-α-Bergamotene | -1.32782 | 0.662837 |
| (*E*)-β-Caryophyllene | -18.8112 | 12.53304 |
| (Z)-3-Hexenol | 6.219171 | 0.902991 |
| (Z)-3-Hexenyl acetate | -0.61611 | 0.409767 |
| (*Z*)-β-Ocimene | 7.02127 | 1.775789 |
| (*Z*,*E*)-α-Farnesene | 11.19305 | -5.56484 |
| 3-Methyl-1-butanol acetate | 2.014565 | 0.709204 |
| α-Amorphene | -7.01628 | 2.898018 |
| α-Cubebene | 3.349275 | -6.71767 |
| α-Gurjunene | -4.62321 | -1.61406 |
| α-Phellandrene | -3.61021 | 6.467417 |
| α-Pinene | 2.511374 | -3.51255 |
| α-Selinene | 8.647261 | 4.098576 |
| Aromadendrene | -4.59631 | -3.58207 |
| β-Elemene | -18.7539 | 9.272236 |
| β-Myrcene | 0.74284 | -7.24785 |
| β-Pinene | -2.81618 | 0.067379 |
| Cadinadiene-1,4 | 1.598506 | 6.953627 |
| Calamenene | 11.09635 | -6.53299 |
| Chamigrene | -0.40436 | 1.126796 |
| Copaene | -6.26678 | 5.285957 |
| δ-Cadinene | -8.28255 | -3.15593 |
| Eucalyptol | -3.56544 | 4.388368 |
| Eudesma-4(14),11-diene | 1.458988 | 0.576601 |
| γ-Terpinene | -3.85518 | 0.825431 |
| Humulene | 14.61323 | -12.8036 |
| Isoledene | 16.13257 | 2.120569 |
| Limonene | 7.872095 | -5.41262 |
| Methyl salicylate | -7.09475 | 3.877746 |
| Nerol | -8.15373 | -2.81148 |
| Nonanal | 0.612197 | 2.793732 |
| Sabinene | 0.380326 | -1.87218 |

**Table S4.** Pairwise comparison of major chemical classes between mānuka paired with broom (MB), heather (MH) or conspecific (MM).

| Chemical class | Treatment | t-value | p-value |
|---|---|---|---|
| Green leaf volatiles | MB vs MH | -0.51 | 0.613 |
| | MB vs MM | 1.92 | 0.063 |
| | MH vs MM | 2.43 | 0.021 |
| Sesquiterpenes | MB vs MH | -1.40 | 0.171 |
| | MB vs MM | 1.82 | 0.078 |
| | MH vs MM | 3.22 | 0.003 |
| Total emissions | MB vs MH | -0.95 | 0.349 |
| | MB vs MM | 1.67 | 0.105 |
| | MH vs MM | 2.62 | 0.013 |

**Table S5.** $\chi^2$ test for *Pyronota festiva* and *Lochmaea suturalis* host-searching behaviour recorded in a Petri dish (df = 1).

| ***Pyronota festiva*** | | | | ***Lochmaea suturalis*** | | | |
|---|---|---|---|---|---|---|---|
| **Treatment** | **Time (h)** | $X^2$ | ***P*** | **Treatment** | **Time (h)** | $X^2$ | ***P*** |
| Mānuka + Blank | 0.25 | 25.07 | <0.001 | Heather + Blank | 0.25 | 20.07 | <0.001 |
| | 0.5 | 29.53 | <0.001 | | 0.5 | 25.07 | <0.001 |
| | 1 | 27.43 | <0.001 | | 1 | 29.93 | <0.001 |
| | 2 | 36.74 | <0.001 | | 2 | 30.63 | <0.001 |
| | 16 | 36.74 | <0.001 | | 16 | 25.36 | <0.001 |
| | 32 | 15.75 | <0.001 | | 32 | 16.71 | <0.001 |
| Heather + Blank | 0.25 | 12.80 | <0.001 | Mānuka + Blank | 0.25 | 5.25 | 0.022 |
| | 0.5 | 25.91 | <0.001 | | 0.5 | 3.54 | 0.060 |
| | 1 | 15.10 | <0.001 | | 1 | 7.12 | 0.008 |
| | 2 | 16.88 | <0.001 | | 2 | 0.39 | 0.531 |
| | 16 | 14.14 | <0.001 | | 16 | 0.00 | 1.000 |
| | 32 | 17.42 | <0.001 | | 32 | 2.41 | 0.121 |
| Mānuka + Heather | 0.25 | 9.08 | 0.003 | Mānuka + Heather | 0.25 | 24.96 | <0.001 |
| | 0.5 | 3.68 | 0.055 | | 0.5 | 14.36 | <0.001 |
| | 1 | 1.23 | 0.267 | | 1 | 17.86 | <0.001 |
| | 2 | 11.74 | 0.001 | | 2 | 23.74 | <0.001 |
| | 16 | 5.81 | 0.016 | | 16 | 27.55 | <0.001 |
| | 32 | 12.68 | <0.001 | | 32 | 21.62 | <0.001 |
